# Biomarker-Based HIV Incidence in a Community Sample of Men Who Have Sex with Men in Paris, France

**DOI:** 10.1371/journal.pone.0039872

**Published:** 2012-06-29

**Authors:** Stéphane Le Vu, Annie Velter, Laurence Meyer, Gilles Peytavin, Jérôme Guinard, Josiane Pillonel, Francis Barin, Caroline Semaille

**Affiliations:** 1 Institut de veille sanitaire, Saint-Maurice, France; 2 Centre for Research in Epidemiology and Population Health, Paris-Sud University, Le Kremlin-Bicêtre, France; 3 APHP, Bichat-Claude Bernard Hospital, Clinical Pharmacokinetic Department, Paris VII Diderot University, Paris, France; 4 National Institute of Health and Medical Research (INSERM), National HIV Reference Center, University Hospital Bretonneau, François Rabelais University, Tours, France; Rollins School of Public Health- Emory University, United States of America

## Abstract

**Background:**

Population-based estimates of HIV incidence in France have revealed that men who have sex with men (MSM) are the most affected population and contribute to nearly half of new infections each year. We sought to estimate HIV incidence among sexually active MSM in Paris gay community social venues.

**Methodology/ Principal Findings:**

A cross-sectional survey was conducted in 2009 in a sample of commercial venues such as bars, saunas and backrooms. We collected a behavioural questionnaire and blood sample. Specimens were tested for HIV infection and positive specimens then tested for recent infection by the enzyme immunoassay for recent HIV-1 infection (EIA-RI). We assessed the presence of antiretroviral therapy among infected individuals to rule out treated patients in the algorithm that determined recent infection. Biomarker-based cross-sectional incidence estimates were calculated. We enrolled 886 MSM participants among which 157 (18%) tested HIV positive. In positive individuals who knew they were infected, 75% of EIA-RI positive results were due to ART. Of 157 HIV positive specimens, 15 were deemed to be recently infected. The overall HIV incidence was estimated at 3.8% person-years(py) [95%CI: 1.5–6.2]. Although differences were not significant, incidence was estimated to be 3.5% py [0.1–6.1] in men having had a negative HIV test in previous year and 4.8% py [0.1–10.6] in men having had their last HIV test more than one year before the survey, or never tested. Incidence was estimated at 4.1% py [0–8.3] in men under 35 years and 2.5% py [0–5.4] in older men.

**Conclusions/ Significance:**

This is the first community-based survey to estimate HIV incidence among MSM in France. It includes ART detection and reveals a high level of HIV transmission in sexually active individuals, despite a high uptake of HIV testing. These data call for effective prevention programs targeting MSM engaged in high-risk behaviours.

## Introduction

Population-based estimates of HIV incidence in France have revealed that incidence rates have declined in all major population groups whereas it has remained steadily high since 2003 in men who have sex with men (MSM) [1]. At the country level, MSM contribute to nearly half of new infections each year. As in many high income countries, these trends are observed against a background of expanding uptake of highly active antiretroviral therapy (HAART) [2], [3].

In 2009, the Prevagay study surveyed a cross-sectional sample of MSM attending commercial gay venues in Paris, which has the largest gay community in France [4]. The collection of blood specimens as part of the study made it possible to identify persons seropositive for HIV and among them those who were recently infected according to a biomarker assay. In the past 15 years laboratory-based methods have been developed to estimate incidence of HIV with a cross-sectional approach [5]–[6]. Still, as with other concurrent approaches to estimate HIV incidence, there are a number of potential sources of error and bias with the biomarker approach [7]. The major problems identified include the selection bias associated with the early testing of recently infected persons as they are recruited in clinics or testing centers [8]; the error associated with an improper estimation of the time spent during recent infection [9]; the presence of “assay non-progressors” patients that remain permanently into the recent infection state [10]; and the perturbation introduced by advanced HIV disease and antiretroviral therapy [11].

Here we report on a biomarker-based estimation of HIV incidence in a sample of sexually active MSM that tries to address these sources of error.

## Methods

### The Prevagay Survey

An anonymous cross-sectional survey (Prevagay) was conducted in 2009 in a sample of 14 commercial venues in Paris gay community such as bars, clubs, bathhouses, backrooms. The methodology and characteristics of study participants have been described previously [4].

In brief, during 56 interventions lasting each 4-hours, male attendees were approached by trained fieldworkers and screened for eligibility, which included being 18 years or older and history of sex with another man in past 12 months. A refusal questionnaire collecting age, self-reported HIV status and reason for refusal was administered to those individuals declining to participate. After giving informed consent, eligible attendees completed a self-administered questionnaire and provided blood sample regardless of self-reported HIV status. Questionnaire data provided information on demographics and behavioral characteristics, comprising self-reported HIV status, history of HIV testing and of sexually transmitted infections. Sample collection consisted in a fingerstick whole blood spotted on a filter paper card. The study protocol was approved by the local Committee for the Protection of Persons and the Data Protection Supervisory Authority.

### Biological Analyses

HIV testing was performed on dried blood spot with a combined immunoassay for detection of both p24 antigen and HIV antibodies (Genscreen ultra HIV Ag-Ab; Biorad) by the National Reference Laboratory for HIV (Tours, France). HIV positive specimens were confirmed by Western blot and then tested for recent infection by the enzyme immunoassay for recent HIV-1 infection (EIA-RI) [12]. The performance of EIA-RI at detecting recently acquired infection had been previously assessed on new diagnoses reported to surveillance [13]. The assay had also been calibrated and used to estimate population-based HIV incidence in France [1]. Its mean RITA duration (the time period from infection during which individuals are classified as positive, indicating a recent infection) was estimated among 952 serial measurements from 298 seroconverters at 0.492 year or 179.7 days [95%CI: 167.2–192.2]. The maximum RITA duration was 2 years. The false recent rate for EIA-RI, that is the proportion of long-term (more than 2 years) infected patients who would test as positive, was determined among 250 chronically infected patients at 0.8% [0–3.1%] [1].

### Detection of Antiretroviral Drugs

It has been shown that prolonged efficient antiretroviral treatment increases the rate of false recent results of the EIA-RI and therefore may lead to overestimate incidence [14]. To rule out treated patients from incidence estimation, we assessed the presence of antiretroviral drugs in EIA-RI positive samples. Concentrations were determined using liquid chromatography coupled with tandem mass spectrometry (UPLC-MS/MS, Acquity UPLC® - Acquity TQD® ) as previously described [15] with a slightly modified extraction for the adaptation to dried blood spots. Since only diagnosed individuals could be on treatment, we tested the presence of antiretroviral drugs among infected men aware of their HIV status and detected as EIA-RI positive.

### Statistical Methods

Because unsufficient quantity of blood impeded to perform drug detection in some sample, multiple imputation by chained equations was used to account for the uncertainty due to missing data regarding use of antiretroviral drugs. HIV incidence rate and its confidence interval were derived according to the estimator described by McWalter and Welte [16].

The number R of recently infected individuals was obtained by considering HIV infected men detected as EIA-RI positive and who did not use antiretroviral treatment. The incidence rate I was estimated by Î = [R- ε(P-R)/(1- ε)]/(Nω) with ω the mean RITA duration, ε the false recent rate, P the number of HIV positive individuals and N the number of negative.

## Results

Of 1578 men invited to participate, 917 (58%) accepted to participate and 886 (56%) both completed the survey questionnaire and provided a blood specimen. Men who refused to participate were not different from those willing to participate in terms of age or self-reported HIV serostatus. Among the 886 MSM participants, 157 (18%) tested HIV positive among which 31 men (20%) were unaware of their HIV infection (table 1).

**Table 1:** HIV testing history according to HIV serostatus and knowledge of it and estimated incidence rate in the Prevagay survey, Paris 2009.

HIV testing history	N	HIV serostatus						Incidence rate [95%CI]	
		Positive				Negative		in person-years	
		Knew they were infected*		Did not know					
Tested in previous year	557	32	(5.7%)	19	(3.4%)	506	(90.8%)	3.5%	[0.1–6.1]
Tested over 1 year ago	274	94	(34.3%)	5	(1.8%)	175	(63.9%)	4.8%	[0.1–10.6]
Never tested	55	0	(0.0%)	7	(12.7%)	48	(87.3%)		
Total	**886**	**126**	**(14.2%)**	**31**	**(3.5%)**	**729**	**(82.3%)**	**3.8%**	[1.5–6.2]

* Persons already diagnosed with HIV at the time of the survey were not supposed to having been tested since their initial diagnosis.

Most men (831/886 = 94%) reported having had an HIV test prior to the survey and (557/886 = 63%) a test within the previous year. Among men already tested, 3% were infected with HIV without knowing it and among men never tested, 13% were found to be HIV-infected.

**Figure 1:**
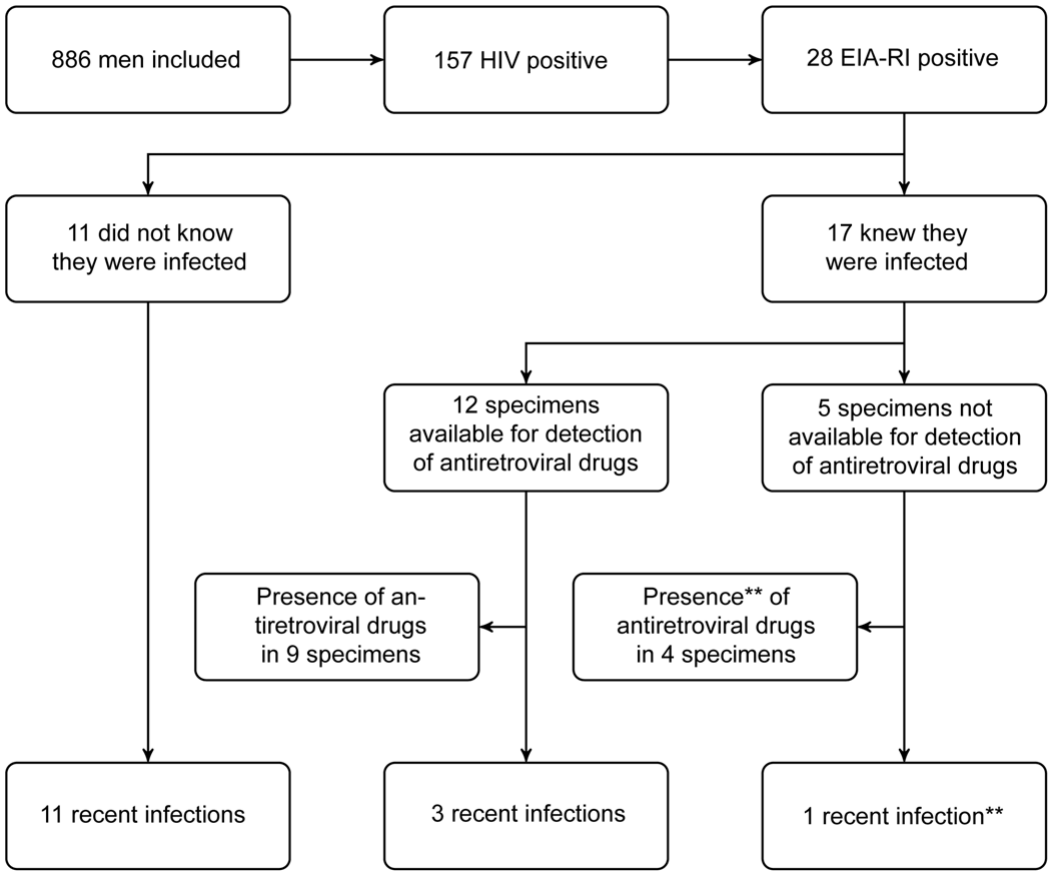
Algorithm used to determine the number of recently infected individuals.* * Recently infected individuals were defined by a EIA-RI positive sample and the absence of antiretroviral treatment ** Average estimates of multiple imputation.

EIA-RI testing indicated a recent infection (EIA-RI positive) for 28 (18%) of the 157 HIV-infected individuals. Respectively, 11 EIA-RI positive results were found among the 31 persons unaware of their HIV infection and 17 among the 126 persons aware of their infection (figure 1).

We detected the presence of antiretroviral drugs in 9 (75%) out of 12 EIA-RI positive specimens from men aware of their infection and who provided sufficient material. The other 5 specimens could not be tested for drug use because of insufficient blood quantity in the paper card. Accounting for the uncertainty due to missing data in these 5 specimens on 100 imputed datasets, we estimated that on average 15 [95%CI: 9–24] infected men out of 157 could be considered as recently infected (EIA-RI positive and free of treatment, cf. figure 1).

The overall HIV incidence was then estimated at 3.8% person-years [95%CI: 1.5–6.2]. It tended to be higher in men under 35 years (4.1% person-years [0–8.3]) or in men having had their last test more than one year before the survey or never tested (4.8% person-years [0.1–10.6]), compared to older men (2.5% person-years [0–5.4]) and men having had a test in previous year (3.5% person-years [0.1–6.1]). Although none of the above differences were statistically significant (p = 0.24 when comparing age groups and p = 0.28 when comparing testing history).

## Discussion

This is the first community-based study to estimate HIV incidence among MSM in France. It reveals a very high level of HIV transmission (3.8% person-years) relative to the estimates we obtained from national case-based surveillance (1% person-years in MSM [1]). While not directly comparable to our estimate, a review of 24 studies reporting incidence estimates in urban MSM from Europe, North America or Australia from 1995 to 2005 found a mean incidence rate of 2.5% person-years [17]. The present study clearly shows that MSM attending gay venues in Paris are at a very high risk of HIV infection with almost 18% prevalence of HIV and a background of high-risk behaviors as reported in the first description of the study: in the previous year, 35% of MSM declared at least one unprotected anal intercourse with a casual partner, 51% had had more than 10 different sex partners, and 18% reported having at least one sexually transmitted infection [4] (a detailed analysis of behavioral results is currently submitted by Velter et al.).

Another important finding from our study is that, although the participants were frequently tested for HIV, as already reported for the MSM population [18], it was not sufficient enough for this population to be timely informed about their HIV status. This has consequences on the ability to adapt personal sexual behaviors and attitudes towards partners. Particularly, in these surveyed venues, our results underline a limitation of serosorting strategies as an alternative to condom use for preventing infection [19].

A major strength of this study is that we could obtain a blood specimen directly in venue sites. This was made possible by dedicating an adequate space for blood draw with venue owners in the preparation of the study. It allowed us to assess the HIV-serostatus of the enrolled subjects, perform the test for recent infection and also exclude treated individuals from the incidence calculation. For this purpose, the use of dried blood spots proved its convenience. Because of practical and financial advantages, dried blood spots are now widely used for the collection of samples for determination of circulating exposures of pharmaceuticals. In the future, they might also be used to detect antiretrovirals in pre/post-exposure prophylaxis in seronegative MSM.

Incidence assays are known to be sensitive to antiretrovirals and the EIA-RI is no exception [14] [11]. In detecting antiretrovirals to exclude potential false recent infection, we controlled this source of error. Conversely, in a largely treated population as is the case in France [20], when excluding treated individuals from the incidence calculation, we could have faced the risk that the remaining group of infected individuals over-represented long term non-progressors since they do not require treatment. It would have therefore increased the risk of misclassification in recent infection determination and thereby the risk of overestimating HIV incidence. However, this level of misclassification due to long term non-progressors is taken into account in the false recent rate, which was determined in a treatment–naive population of chronically infected individuals [1].

Another asset of the study relates to its cross-sectional design in venues which allowed us to enrol and test individuals independently of their time since HIV infection. As opposed to recruitments of MSM in testing facilities, where persons may be more likely to seek a test as a consequence of a recent risk or symptoms of primary HIV infection or other sexually transmitted infections, our incidence determination is not prone to such a test-seeking bias [8], [21].

The representativity of our study population must however be discussed. The acceptance rate we obtained (58%) is comparable to those obtained in similar cross-sectional collection of behavioral data and biological samples in gay venues in Europe and Australia [22]–[26]. However, only men who attended the surveyed venues had the opportunity to participate. As a consequence, the estimates for incidence can not apply for all MSM in Paris. As there were no noticeable difference between men who participate and those who refused, and also no significant difference between participants in the Prevagay survey and those of a larger behavioral survey conducted in 72 commercial venues in Paris [27], our sample is hopefully representative of the venue population.

In most industrialized countries, MSM are the most affected population by HIV infection and incidence estimation at the community level is crucial. Our study provides an example of using biomarker-based methodology to estimate HIV incidence in gay venues that includes the detection of antiretroviral drugs in dried fluid spots. In the context of wide use of HAART, new preventive strategies might not only expand their therapeutic use but may also expand the use among negative as a prophylactic strategy [28]. In that perspective, we emphasize the need to include the detection of antiretroviral drugs in the algorithm to detect recent infection.

With a detailed view of the level of HIV transmission and testing behaviours among sexually active MSM in Paris, our study reveals a high level of HIV incidence in the context of gay venues and that one can not only rely on an intensive testing policy to control the epidemics. These data also call for effective prevention programs targeting MSM engaged in high risk behaviours.
